# Matrix-Assisted Laser Desorption Ionization - Time of Flight Mass Spectrometry: An Emerging Tool for the Rapid Identification of Mosquito Vectors

**DOI:** 10.1371/journal.pone.0072380

**Published:** 2013-08-15

**Authors:** Amina Yssouf, Cristina Socolovschi, Christophe Flaudrops, Mamadou Ousmane Ndiath, Seynabou Sougoufara, Jean-Sebastien Dehecq, Guillaume Lacour, Jean-Michel Berenger, Cheikh Sadibou Sokhna, Didier Raoult, Philippe Parola

**Affiliations:** 1 Aix Marseille Université, Unité de Recherche en Maladies Infectieuses et Tropicales Emergentes (URMITE), UM63, CNRS 7278, IRD 198 (Dakar, Sénégal), Inserm 1095, Marseille, France; 2 APHM, CHU Timone, Pôle Infectieux, Marseille, France; 3 Entente Interdépartementale de Démoustication Méditerranée, Montpellier, France; 4 Agence Régionale de Santé (ARS) Océan Indien, Saint-Denis, France; New Mexico State University, United States of America

## Abstract

**Background:**

The identification of mosquito vectors is typically based on morphological characteristics using morphological keys of determination, which requires entomological expertise and training. The use of protein profiling by matrix-assisted laser desorption/ionization time-of-flight mass spectrometry (MALDI-TOF-MS), which is increasingly being used for the routine identification of bacteria, has recently emerged for arthropod identification.

**Methods:**

To investigate the usefulness of MALDI-TOF-MS as a mosquito identification tool, we tested protein extracts made from mosquito legs to create a database of reference spectra. The database included a total of 129 laboratory-reared and field-caught mosquito specimens consisting of 20 species, including 4 *Aedes* spp., 9 *Anopheles* spp., 4 *Culex* spp., *Lutzia tigripes*, *Orthopodomyia reunionensis* and *Mansonia uniformis*. For the validation study, blind tests were performed with 76 specimens consisting of 1 to 4 individuals per species. A cluster analysis was carried out using the MALDI-Biotyper and some spectra from all mosquito species tested.

**Results:**

Biomarker mass sets containing 22 and 43 masses have been detected from 100 specimens of the *Anopheles*, *Aedes* and *Culex* species. By carrying out 3 blind tests, we achieved the identification of mosquito vectors at the species level, including the differentiation of *An. gambiae* complex, which is possible using MALDI-TOF-MS with 1.8 as the cut-off identification score. A cluster analysis performed with all available mosquito species showed that MALDI-Biotyper can distinguish between specimens at the subspecies level, as demonstrated for *An gambiae* M and S, but this method cannot yet be considered a reliable tool for the phylogenetic study of mosquito species.

**Conclusions:**

We confirmed that even without any specific expertise, MALDI-TOF-MS profiling of mosquito leg protein extracts can be used for the rapid identification of mosquito vectors. Therefore, MALDI-TOF-MS is an alternative, efficient and inexpensive tool that can accurately identify mosquitoes collected in the field during entomological surveys.

## Introduction

Over the last few decades, numerous mosquito-borne infections have emerged or reemerged throughout the world. The medical importance and burden of these infections is enormous [1]. They are not limited to tropical areas, where malaria, dengue, and Chikungunya are well known threats to the local population and returning travelers [1], [2]. Some diseases, such as West Nile disease, are spreading geographically, and their frequency of incidence is increasing [3]. Moreover, the recent outbreak of the mosquito-borne Chikungunya virus in the Indian Ocean Islands and India, which has since spread throughout many tropical countries and even reached Europe in 2007, illustrates the current medical importance of the globalization of vector-transmitted infections [4].

With the growing importance of mosquito-borne diseases, entomological surveys, including the collection and identification of mosquitoes, are needed to better understand transmission dynamics [5]. These surveys are essential in the control of vector-borne diseases because they provide information about the vector species involved in the transmission, which is essential for planning effective control measures and monitoring their impact [6]. Species identification is the first step in entomological studies [6], and misidentification can have negative impacts on public health [7].

Mosquitoes can be identified morphologically at the family, genus, and species levels using the numerous taxonomic keys available from different regions of the world [8]. However, identification remains difficult due mainly to specimen damage during collection or interspecies similarity within a species complex [9], [10]. In addition, there is a worldwide decrease in the availability of systematic experts [11].

Molecular methods are currently being developed to identify arthropods by mitochondrial sequencing of the nuclear internal transcribed spacer 2 (ITS2) [7], [9], [10] or IGS regions of rDNA [12]–[14]. These methods appear to be promising and are increasingly being used for the classification of complex species. Therefore, several polymerase chain reaction (PCR) methods have been developed as identification tools for mosquitoes, specifically *Anopheles* [10], [14], *Aedes* [15], and *Culex* species [9]. Despite its specificity, reproducibility and sensitivity, this technique remains time-consuming, as it requires several mosquito specimen processing steps that are technically demanding and expensive. Additionally, this method requires sequence information about a chosen gene prior to PCR and cannot be easily applied to the rapid identification and classification of specimens [16].

Protein profiling using matrix-assisted laser desorption/ionization time-of-flight mass spectrometry (MALDI-TOF-MS) was developed during the last decade as an important tool for the identification and phylogenetic classification of microorganisms [16]–[18]. An evaluation of the MALDI-TOF-MS-based detection of bacteria showed that the results of mass spectrometry-based identification were consistent with those of 16S rRNA sequencing [17], [19]. MALDI-TOF-MS protein profiling has been used to accurately identify unicellular Eukarya [20], Archaea organisms [21] and giant viruses [22], and it has been added to the species classification tool kit as a complementary method to DNA sequencing [21].

MALDI-TOF-MS analysis of protein extracts from insects such as *Drosophila* showed that the spectra generated were reproducible for each species [23], [24]. In addition, the analysis of 3 species of aphids (insects that feed on plants) using this method revealed species-specific protein profiles that were independent of the diet of each species and could be used to differentiate between the species [25]. In 2011, the MALDI-TOF-MS-based identification of 2 *Culicoides* species showed that MALDI-TOF MS analyses of specimens without the abdomen were consistent, independent of the sex and age of the specimens [26]. Another study of 14 *Culicoides* species showed that fresh specimens yield consistent spectra profiles and that the legs, head and wings could be used for morphological identification. The results obtained by a blind test of 111 field-caught specimens that were analyzed using available databases of *Culicoides* species confirmed the use of MALDI-TOF-MS as an alternative tool to identify field-caught biting midges [27]. The MALDI-TOF-MS approach was first used for the entomological survey of *Culicoides* biting midges [28]. Recently, the accurate identification of ticks by MALDI-TOF-MS, from either the whole specimen [29] or the legs only [30], has been reported. It has also been reported that MALDI-TOF-MS spectra could be used to age grade *Anopheles gambiae* female mosquitoes with greater accuracy than other available methods [31]. Finally, MALDI-TOF-MS spectra have been shown to be able to accurately identify closely related mosquito species of the genus *Anopheles* using the thorax and head for protein extraction [32].

The aim of this study was to use MALDI-TOF-MS on protein extracts from mosquito legs to identify mosquitoes from different genera and species. The goal was to establish a reference sequence database for mosquitoes and to perform blind tests to evaluate the accuracy of the MALDI-TOF-MS methodology in species identification.

## Materials and Methods

### Specimens used to create a database

**Figure 1:**
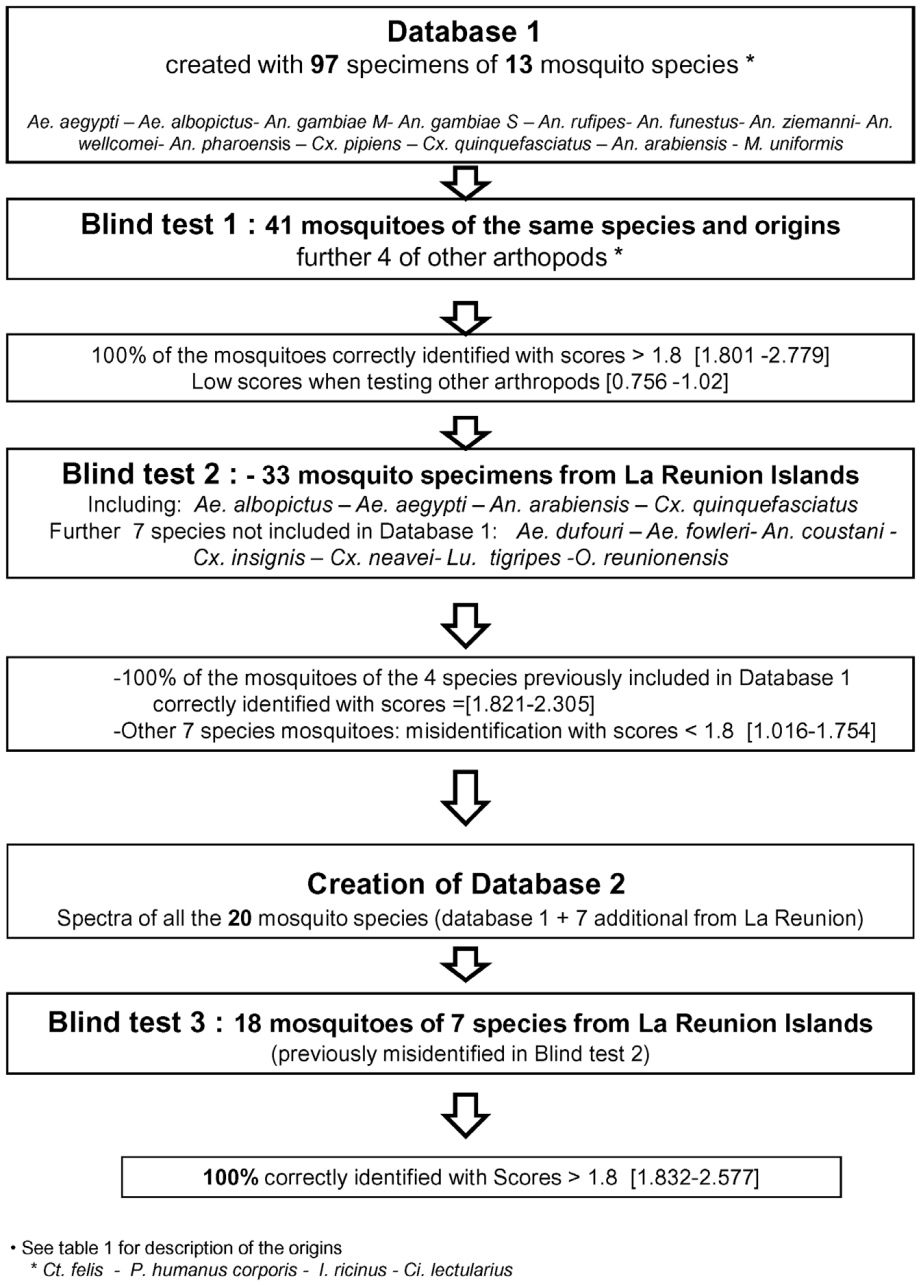
Study design to evaluate the potential of MALDI-TOF-MS for identifying mosquito vectors.

The first database (database 1) was created using non-engorged, fresh laboratory-reared mosquitoes of various origins and engorged fresh mosquitoes collected in the field in Senegal. The laboratory mosquitoes used were adults of the following species: *Culex quinquefasciatus*, *Aedes aegypti*, *Culex pipiens*, *Ae. albopictus*, and *Anopheles gambiae* molecular form M and S (Table 1). The adult mosquitoes that were caught in the field in Senegal included *An. funestus, An. ziemanni, An. arabiensis, An. wellcomei, An. rufipes, An. pharoensis* and *Mansonia uniformis*. These mosquitoes were collected using human landing catches (HLCs), CDC light traps and indoor resting catches by aspiration. The specimens were identified morphologically using morphologic identification keys [33]. Almost all specimens tested were female. The morphological identification of *An. arabiensis* (a member of the *An. gambiae* complex) was confirmed by a PCR-restriction fragment length polymorphism (PCR-RFLP) analysis of part of the 28S coding sequence and the IGS regions of the rDNA [12]. Specimens of other species were collected from Reunion Island, a French territory in the Indian Ocean, and these specimens were used to create a larger database (database 2) that included *Ae. albopictus, Ae. aegypti, An. arabiensis, Cx. quinquefasciatus*, and 7 additional species not included in database 1: *Ae. dufouri, Ae. fowleri, An. coustani, Cx. insignis, Cx. neavei, Lutzia tigripes*, and *Orthopodomyia reunionensis* (Table 1). A flow chart of the study is summarized in Figure 1.

**Table 1:** Mosquito species used to establish a MALDI-TOF MS reference database.

Species	Number	Geographical origin	Source
1. *An. gambiae* form S	6F	Kenya	Lab. IRD Montpellier
2. *Ae. albopictus*	9F	France	Lab. EID
3. *Ae. aegypti*	12F/9F	Bora-Bora/Mayotte	Lab. EID
4. *Cx. quinquefasciatus*	6F	USA	Lab. EID
5. *Cx. pipiens*	7F	France	Lab. EID
*6. An. gambiae* form M	7F	Senegal	Lab. IRD Senegal
7. *An. funestus*	5F	Senegal	Field Senegal*
8. *An. arabiensis*	6F	Senegal	Field Senegal**
9. *An. wellcomei*	6F	Senegal	Field Senegal*
10. *An. ziemanni*	6F	Senegal	Field Senegal*
11. *An. pharoensis*	5F	Senegal	Field Senegal*
12. *An. rufipes*	5F	Senegal	Field Senegal*
13. *M. uniformis*	6F	Senegal	Field Senegal*
14. *An. coustani*	3F; 1M	Reunion	Field Reunion**
15. *Ae. dufouri*	5F	Reunion	Field Reunion**
16. *Ae. fowleri*	5F	Reunion	Field Reunion**
17. *Cx. neavei*	5F	Reunion	Field Reunion**
18. *Cx. insignis*	5F	Reunion	Field Reunion**
19. *Lu. tigripes*	5F	Reunion	Field Reunion**
20. *O. reunionensis*	5F	Reunion	Field Reunion**
**Total specimens**	**129**	**-**	-

[1]–[13] =  Database 1 species. [1]–[20] =  Database 2 species.

* Collection of adults.

** Collection of larvae and emergence of adults in laboratory (M: male; F: female).

Lab =  Laboratory.

### MALDI-TOF-MS procedures

#### Preparation of samples for MALDI-TOF-MS

All of the legs from each mosquito were homogenized manually in 20 µl of 70% formic acid and 20 µl of 50% acetonitrile in 1.5 ml microtubes using pellet pestles (Fischer Scientific, Strasbourg, France). The homogenates were centrifuged at 10,000 rpm for 20 s, and 1 µl of the supernatant of each sample was deposited on a steel target plate (Bruker Daltonics™, Wissembourg, France) into four spots for each sample [30]. Then, 1 µL of CHCA matrix composed of saturated α-cyano-4-hydroxycynnamic acid (Sigma®, Lyon. France), 50% acetonitrile, 2.5% trifluoroacetic acid and HPLC-grade water was directly overlaid on each sample on the target plate, dried for several minutes at room temperature and introduced into the MALDI-TOF-MS instrument for analysis.

#### MALDI-TOF-MS parameters

Protein mass profiles were obtained using Microflex LT MALDI-TOF Mass Spectrometry (Bruker Daltonics machine) with Flex Control software (Bruker Daltonics). We realized measurements in the linear positive-ion mode [34] within a mass range of 2–20 kDa. Each spectrum corresponds to ions obtained from 240 laser shots performed in six regions of the same spot. The spectrum profiles obtained were visualized with Flex analysis 3.3 software and exported to the MALDI-Biotyper v. 3.0.

#### Spectra analysis and reference database creation

An evaluation of species spectra reproducibility was performed by comparing the average spectra obtained from the four spectra of each specimen within a species using the ClinProTools 2.2 software (Bruker Daltonics). Then, for each species, the spectra from at least 5 specimens were exported to MALDI-Biotyper 3.0 to create database 1 (Table 1).

#### MALDI-TOF-MS biomarker mass sets

To determine the species differential peaks from the samples of 9 *Anopheles* spp, 4 *Culex* spp. and 4 *Aedes* spp. tested (Table 1), all spectra of the specimens from each species were loaded into ClinProTools software to generate a peak list for each species in the 2 to 20 kDa Mass range. The peak lists with intensity values were exported to Excel files for data analysis. The peaks with a relative intensity below 2% were excluded from the lists. Different software parameters were set to the following values for spectra preparation: Noise threshold  =  2.00; Maximal peak shift  =  800 ppm and Match to calibrant peaks  =  10%. For the peak calculation, the signal to noise threshold was 4.00 with an aggregation of 800 ppm.

### Blind tests for study validation

#### Blind test 1

The reference spectra of each species were compared to evaluate the database. A blind test was then performed using new adult specimens from our laboratory-reared colonies and field-caught mosquitoes from Senegal that had corresponding reference spectra in our database (figure 1). For each species, 1 to 4 new specimens were used. Both molecular forms of *An. gambiae* (M and S) were carefully evaluated to identify a match with the corresponding database. We also tested 2 specimens of different arthropod species that were not included in our database, including ticks (*Ixodes ricinus*), fleas (*Ctenocephalides felis*), lice (*Pediculus humanus corporis*) and bed bugs (*Cimex lectularius*). The results are presented in the MALDI-Biotyper software as Log Score values that correspond to a matched degree of signal intensities of mass spectra of the query and the reference spectra, and these score values for species identification were obtained for each spectrum of the tested samples [35].

#### Blind test 2

A second blind test was later performed as described above with specimens of 11 species collected from Reunion Island, including 7 species that had no corresponding reference spectra in database 1 (Figure 1). The spectra of 4 to 5 specimens per species were obtained and compared with database 1. A cut-off score for accurate identification was established. The new spectra of the 7 new species from Reunion Island were added to database 1 to create database 2, consisting of a total of 20 reference mosquito species.

#### Blind test 3

New mosquito specimens corresponding to the 11 species collected in Reunion Island were tested against database 2, with at least 1 to 4 specimens per species. A score value was obtained for each mosquito specimen tested.

### Cluster analysis

The MSP dendrogram function of the MALDI-Biotyper 3.0 shows how organisms are related to one another. The MSP dendrogram compares several spectra and clusters them according to the protein mass profile, i.e., their mass signals and intensities [17], but not by protein identity. We performed hierarchical clustering of the mass spectra of all tested species that were loaded in the final database using the MSP dendrogram function. The objective was to determine whether this method could be used to cluster the different mosquito species.

### Ethical approval

The Senegalese collectors gave prior informed written consent. Permission was obtained from residents to collect samples in their rooms. This study was approved by the National Ethical Committee of Senegal (Health ministry). The field studies did not involve endangered or protected species. Specific permission was not required for specimens collected from Reunion Island.

## Results

### Spectra analysis/initial reference database creation

**Figure 2:**
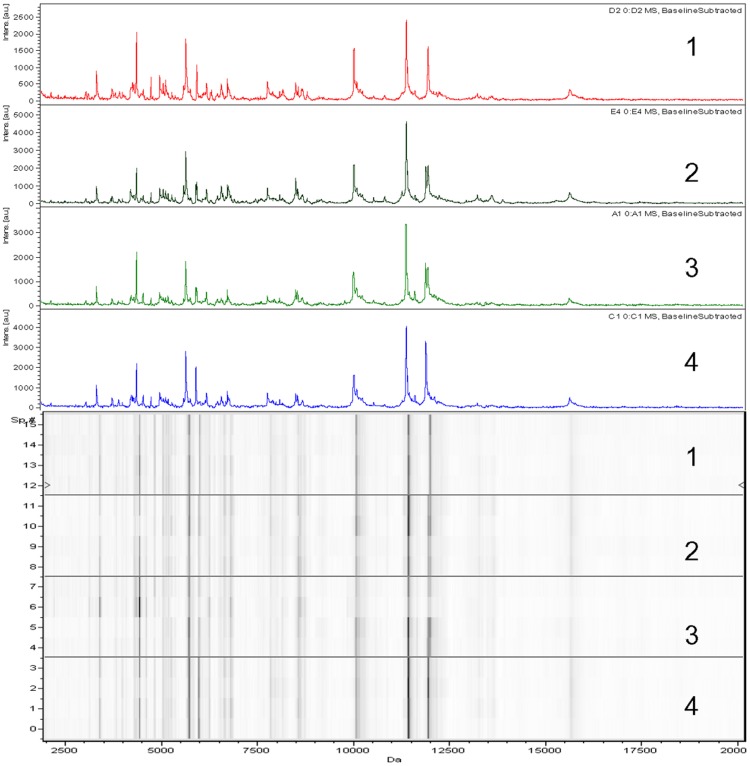
MALDI-TOF-MS spectra obtained from leg protein extraction of four *An.* *gambiae* form M specimens.

A total of 95 specimens, including 13 mosquito species with at least 5 specimens per species, were subjected to MALDI-TOF-MS analysis to create database 1 (Table 1). The MALDI-TOF-MS analysis of the protein extracts prepared from mosquito legs showed spectra with peaks of high intensities in the range of 2–20 kDa. The quality of spectra and intensity of spectral peaks was consistent in all species tested (Figure 2). Using a Flex analysis, we observed that the protein spectra profiles obtained from all mosquito species were similar between species. In specimens of the same species, the major protein peaks appeared in each spectrum. The alignment of spectra from different specimens using the ClinProTools 2.2 software confirmed the reproducibility of the spectra (Figure 2). Based on these results, the spectra of these 13 species were loaded to create database 1.

### MALDI-TOF MS biomarker mass sets

**Figure 3:**
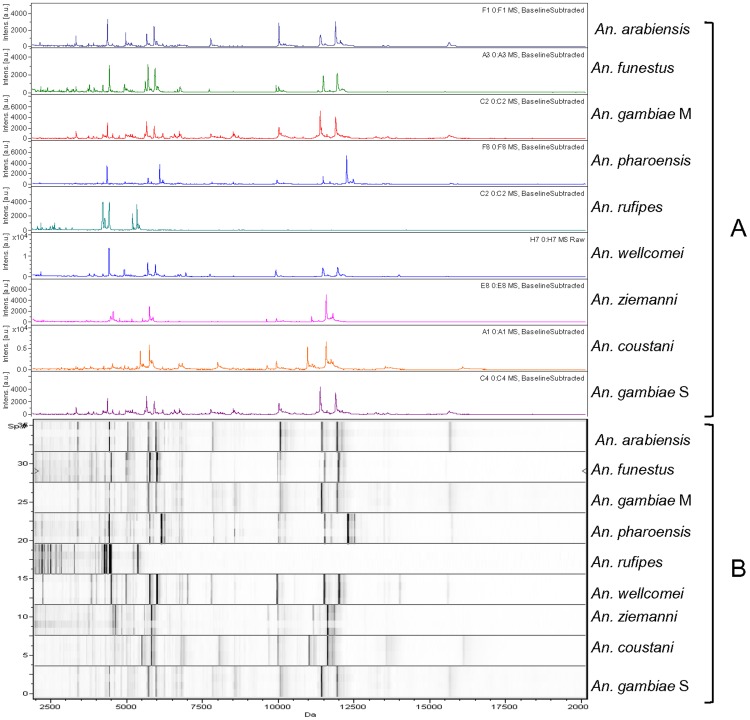
Matrix-assisted laser desorption time of flight mass spectra profiles of legs only from *Anopheles* species. View of spectra profiles using Flex analysis (A) and ClinProTools software (B).

The alignment of spectra profiles of different species from each genus in Flex analysis and ClinProTools revealed the repetition of some peaks (Figure 3). Thus, we determined the average peaks of 100 mosquito specimens, including 9 *Anopheles* spp., 4 *Aedes* spp. and 4 *Culex* spp. in the mass range of 2–20 kDa. In 53 samples of *Anopheles* spp., we detected 174 peaks and a total of 22 specific biomarker masses distinguishing the 9 species (Table 2). In 23 samples of *Culex* spp., 139 peaks were detected on average, with 24 biomarker masses differentiating the species (Table 3). Of the 24 samples of *Aedes* spp., 151 peaks were detected on average, and 43 biomarker masses differentiated the species (Table 4).

**Table 2:** Potential biomarker masses for the 9 *Anopheles* species included in database 2.

Biomarker mass	*An. arabiensis*	*An. funestus*	*An. gambiae* M	*An. pharoensis*	*An.* *rufipes*	*An. wellcomei*	*An. ziemanni*	*An. coustani*	*An. gambiae* S
3689.42	No	**Yes**	No	No	No	No	No	No	No
5416.96	No	No	No	No	No	No	No	**Yes**	No
5462.7	No	No	No	No	No	**Yes**	No	No	No
6896.04	No	No	No	No	No	No	No	**Yes**	No
7531.63	No	**Yes**	No	No	No	No	No	No	No
7771.45	No	**Yes**	No	No	No	No	No	No	No
8062.03	No	No	No	No	No	No	No	**Yes**	No
8603	No	No	No	No	No	No	No	No	**Yes**
9674.33	No	No	No	No	No	No	**Yes**	No	No
10427.51	**Yes**	No	No	No	No	No	No	No	No
10583.37	No	No	No	No	No	No	No	No	**Yes**
10869.76	No	No	No	No	No	No	No	No	**Yes**
11020.41	No	No	No	No	No	No	No	**Yes**	No
11325.26	No	No	No	No	No	No	No	No	**Yes**
12519.88	No	No	No	**Yes**	No	No	No	No	No
12844.78	**Yes**	No	No	No	No	No	No	No	No
12986.02	**Yes**	No	No	No	No	No	No	No	No
13272.58	No	No	No	No	No	No	No	No	**Yes**
13495.81	No	No	No	**Yes**	No	No	No	No	No
13633.11	No	No	No	No	No	No	No	No	**Yes**
13654.49	No	No	No	No	No	No	No	No	**Yes**
13671.98	No	No	No	No	No	No	No	No	**Yes**
**Total**	**3**	**3**	**0**	**2**	**0**	**1**	**1**	**4**	**8**

**Table 3:** Potential biomarker masses for the 4 *Culex* species included in database 2.

Biomarker Mass	*Cx. neavei*	*Cx. insignis*	*Cx. pipiens*	*Cx. quinquefasciatus*
2156.11	**Yes**	No	No	No
5347.49	No	**Yes**	No	No
6290.27	No	No	No	**Yes**
6564.69	No	**Yes**	No	No
6724	No	**Yes**	No	No
6718.04	No	**Yes**	No	No
6984.93	No	**Yes**	No	No
7224.5	No	**Yes**	No	No
7729.26	No	**Yes**	No	No
7744.19	No	**Yes**	No	No
7366.32	No	No	No	**Yes**
7408.48	No	No	No	**Yes**
8813.04	No	**Yes**	No	No
10687.97	No	**Yes**	No	No
10822.8	No	**Yes**	No	No
12181.35	No	No	No	**Yes**
13180.86	No	No	No	**Yes**
13356.52	No	**Yes**	No	No
13773.04	No	**Yes**	No	No
14919.74	No	No	No	**Yes**
14940.15	No	No	No	**Yes**
15058.84	No	No	No	**Yes**
15325.47	No	**Yes**	No	No
15479.87	No	**Yes**	No	No
**Total**	**1**	**15**	**0**	**8**

**Table 4:** Potential biomarker masses for the 4 *Aedes* species included in database 2.

Biomarker mass	*Ae. aegypti*	*Ae. albopictus*	*Ae. fowleri*	*Ae. dufouri*
2242.68	**Yes**	No	No	No
2495.49	**Yes**	No	No	No
2529.54	No	**Yes**	No	No
2554.47	No	No	**Yes**	No
2707.99	No	No	**Yes**	No
3136.3	No	**Yes**	No	No
3254.5	No	**Yes**	No	No
3787.81	No	No	**Yes**	No
4119.12	No	No	No	**Yes**
5022.41	**Yes**	No	No	No
5155.49	**Yes**	No	No	No
5313.11	No	No	**Yes**	No
6214.63	No	No	No	**Yes**
6345.41	No	No	No	**Yes**
6494.04	No	No	No	**Yes**
7281.55	No	No	No	**Yes**
7358.94	No	No	No	**Yes**
7403.29	No	**Yes**	No	No
7542.16	**Yes**	No	No	No
7770.22	No	**Yes**	No	No
7860.24	No	**Yes**	No	No
8081.46	No	No	**Yes**	No
8696.01	**Yes**	No	No	No
8666.01	No	**Yes**	No	No
8729.7	**Yes**	No	No	No
8763.38	No	No	**Yes**	No
9155.96	**Yes**	No	No	No
9449.18	No	No	No	**Yes**
9953.46	No	**Yes**	No	No
10168.78	No	**Yes**	No	No
10198.59	No	No	No	**Yes**
10275.59	No	No	No	**Yes**
10300.35	No	No	**Yes**	No
10776.35	**Yes**	No	No	No
11085.42	No	No	**Yes**	No
11602.66	No	**Yes**	No	No
12856.15	No	No	No	**Yes**
14562.15	No	No	No	**Yes**
14716.64	No	No	No	**Yes**
14856.44	**Yes**	No	No	No
15014.31	**Yes**	No	No	No
15138.93	No	No	**Yes**	No
18138.67	**Yes**	No	No	No
**Total**	**12**	**10**	**9**	**12**

### Blind tests

Querying the spectra using the databases yielded satisfactory results, with identification score values between 2.122 and 2.714.

#### Blind test 1

A total of 41 adult specimens, consisting of 2 to 4 specimens for each of the 13 species, were tested against database 1. A comparison of all these samples in the database using the MALDI-Biotyper software revealed satisfactory results because all 41 adult specimens tested (4 spots per specimen) led to the correct identification at the species level with high identification scores (between 1.801 to 2.779) (Table 5). Only three out of 13 species, including *An. funestus*, *An. ziemanni* and *An. wellcome*i, were identified with scores lower than 2. Interestingly, it was possible to discriminate both molecular forms (S and M) within the *An. gambiae* sample set in which 100% of the individuals tested matched with their corresponding forms. The spectra of other arthropod groups, such as the louse, bed bug, flea and tick, which are not in the database, scored lower (between 0.41 and 1.02).

**Table 5:** Mosquito species used to evaluate the established MALDI-TOF-MS reference database and score value results for all samples identified in the 3 blind tests performed.

Species	Number tested	Source	ID score values [Low-High]
*An. gambiae* form S	3	Laboratory IRD Montpellier	[1.872–2.609]
*An. gambiae* form M	3	Laboratory IRD Senegal	[2.067–2.779]
*An. funestus*	2	Field Senegal	[1.83–1.945]
*An arabiensis*	6	Field Senegal/Reunion	[1.97–2.452]
*An. wellcomei*	2	Field Senegal	[1.82–1.882]
*An. pharoensis*	2	Field Senegal	[2.213–2.254]
*An. rufipes*	3	Field Senegal	[2.04–2.308]
*An. ziemanni*	3	Field Senegal	[1.83–1.990]
*An. coustani*	1	Field La Reunion	2.294
*Ae. albopictus*	7	Field Marseille/Reunion	[2.127–2.305]
*Ae. aegypti*	8	Field Senegal/Reunion	[1.881–2.355]
*Ae. dufouri*	3	Field Reunion	[2.335–2.577]
*Ae. fowleri*	3	Field Réunion	[2.253–2.365]
*Cx. quinquefasciatus*	8	Field Senegal/Reunion	[1.821–2,183]
*Cx. pipiens*	3	Laboratory and field France	[1.949–2.024]
*Cx. neavei*	3	Field Reunion	[1.986–2.409]
*Cx. insignis*	3	Field Reunion	[2.172–2.425]
*Lu. tigripes*	3	Field Reunion	[2.256–2.39]
*M. uniformis*	4	Field Senegal	[1.871–2,518]
*O. reunionensis*	2	Field Reunion	[2.325–2.358]
**Other arthropods**			
*Ct. felis*	1	URMITE Laboratory colonies	0.981
*P. humanus corporis*	1	URMITE Laboratory colonies	0.756
*I. ricinus*	1	URMITE Laboratory colonies	0.829
*Ci. Lectularius*	1	URMITE Laboratory colonies	1.02
**Total tested**	**76**	-	-

#### Blind test 2

Out of the 33 specimens, consisting of 11 species from Reunion Island, 4 species were accurately identified from database 1 with score values between 1.821 and 2.305. The other 7 species without corresponding spectra in the database matched incongruently with spectra in database 1 but only with low score values (between 0.606 and 1.754). The spectra of 34 new specimens corresponding to these 7 species were then added to database 1 to build database 2, which includes 129 reference spectra from 20 species.

#### Blind test 3

A total of 18 specimens of 7 species from Reunion Island that were not identified in blind test 2 were evaluated against database 2. The results were satisfactory because all of the specimens were correctly identified with identification score values between 1.82 and 2.779 (Table 5). On average, in the three tests, the mosquito specimens were identified with a score of 2.165 with a maximal score of 2.779 and a minimal score of 1.82.

### Cluster analysis

**Figure 4:**
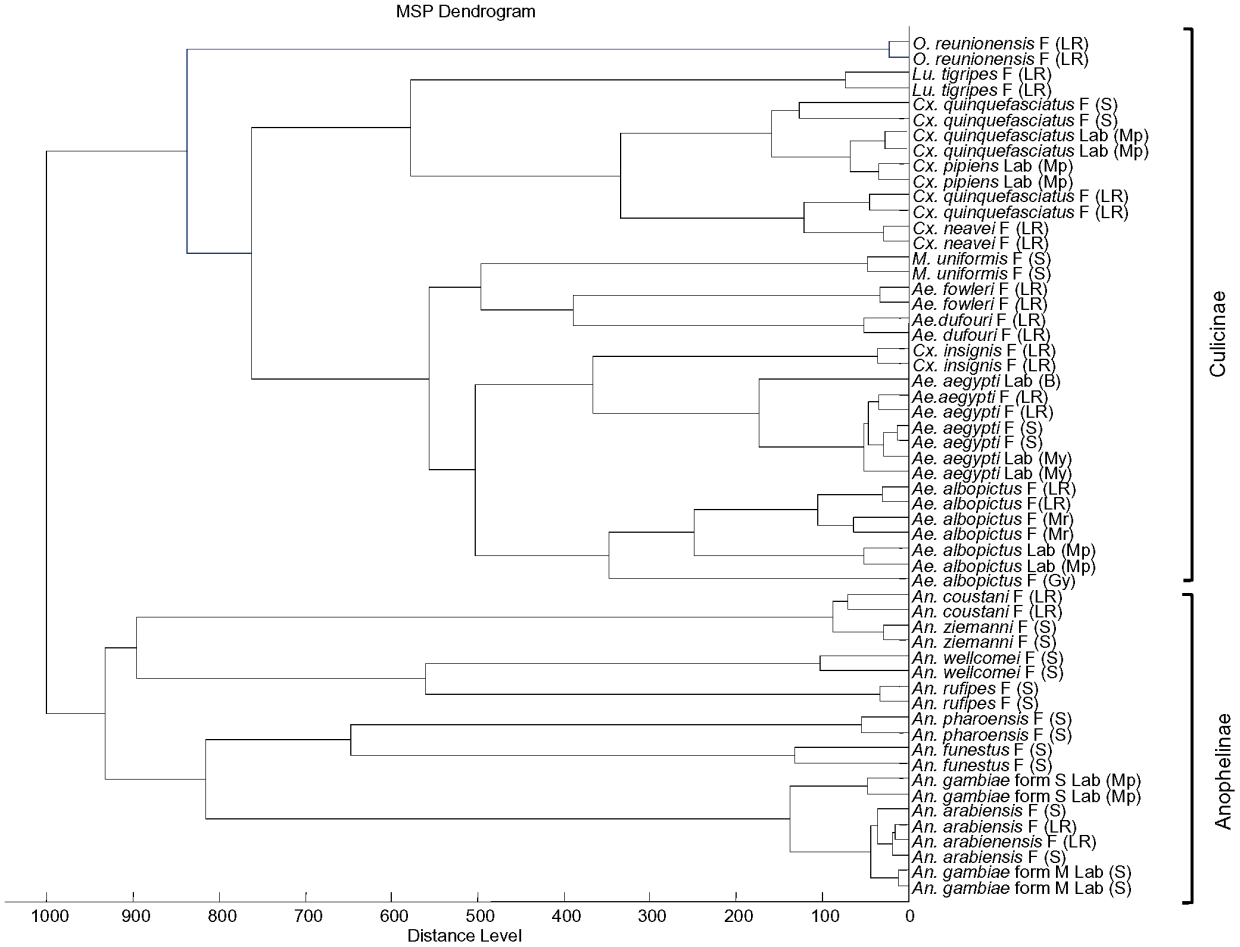
Dendrogram obtained by a cluster analysis of the spectra obtained from laboratory-reared and field-collected mosquitoes. The mosquitoes were clustered using the MALDI Biotyper software.

MSP spectra from mosquito specimens were used to generate a dendrogram with the aim to cluster all mosquito species present in database 2 according to their spectra. The dendrogram obtained is shown in Figure 4. The clusters formed were consistent with species and genera classifications, with the exception of 2 specimens of *Cx. insignis* that formed a cluster within *Ae. aegypti*.

## Discussion

The mosquitoes evaluated in this study are potential vectors of several infectious diseases that are prevalent in tropical and/or temperate areas of the world. We chose to use only legs to avoid potential traces of remaining blood meals in the spectra [27], [32]. Using the ClinProTools software of MALDI-TOF-MS, we generated biomarker masses from 22 to 43 that are similar to those obtained from *Culicoides* biting midges [27]. Thus, the 155 peaks detected for the three mosquito genera are higher than the number of peaks obtained for cryptic *Anopheles gambiae* complex but lower than those obtained for *Culicoides* biting midges [32]. The biomarker mass detection proved that MALDI-TOF-MS are a suitable tool for mosquito species identification. Furthermore, we can identify the most abundant proteins in different mosquito species by tandem mass spectrometry, but these results could not be linked with the identified peaks detected by MALDI-TOF. When the proteins are ionized in the MALDI-TOF source, all the proteins are competing to capture protons (i.e., to be ionized). In the MALDI spectrum, we do not detect the most abundant proteins; rather, we detect the proteins with the highest ionization capability. If we alter the mixture of proteins used to obtain the MALDI spectrum (e.g., if we compare two species of mosquito), we may observe a gain or loss of peaks between the two species of mosquito because even though the proteins are present in the two conditions, they are not necessarily ionized.

We have shown that MALDI-TOF-MS analysis of protein extracts of mosquito legs is a suitable technique for identifying mosquitoes using a reference database. The results obtained using this method corroborated the results obtained using morphological methods. The validity of the database was established by a blind test in which 100% of the specimens were correctly identified by MALDI-TOF-MS when the corresponding reference spectra were available in the database. These specimens had high identification scores (71% had scores > 2 and 29% had scores between 1.8–1.9, thus 100% had scores > 1.8). When identifying bacteria by the MALDI-TOF-MS approach, a score of 1.8 generally indicates the reliable identification of bacterial genera [16], [17].

For specimens without corresponding species reference spectra in the database, such as the 7 mosquito species from Reunion Island evaluated against database 1 that contained reference spectra of other mosquito species from Senegal, some mismatch with reference spectra was observed, but these identification scores were lower (< 1.8). These results allowed us to set an identification score cut-off value of 1.8, thereby facilitating a more accurate and definitive identification of the mosquito species. Interestingly, we were also able to distinguish between the two molecular forms of *An. gambiae*, S and M, that belong to the same species but have a different ecological distribution, from Kenya and Senegal, respectively, and different susceptibility to infection by *Plasmodium* spp. [36]–[38].

Because reliable MALDI-TOF-MS results were obtained for the identification of ticks using leg extract samples, we also used legs for mosquito identification [30]. The use of legs for identification allows the preservation of the remainder of the body for other purposes, such as pathogen detection in the salivary gland or stomach and the study of parity in the ovaries [6].

The MALDI-Biotyper software cannot yet be considered to be a reliable tool for studying the phylogeny of mosquitoes. Using the MSP dendrogram function of MALDI-Biotyper 3.0, 20 mosquito species in our database clustered according to species and sometimes according to strain (Figure 4), which is similar to the previously performed cluster analysis of *Culicoides* species [27] or 16S rRNA sequencing in bacteria [16]. One species of genus *Culex* (*C. insignis*) clustered with genus *Aedes,* but such incoherence has also been observed in tick classification studies [29], [30], as well as *Anopheles* species [32].

## Conclusions and Perspectives

The rapid identification of mosquito species will contribute to the design and implementation of effective prevention measures for mosquito-borne diseases. It will also contribute to studies of vector biology, including the evolution of the dispersion of the vector population, such as that of *Ae. albopictus*, around the world [39], [40] and the risk of disease transmission. The method evaluated in this study presents several advantages, including rapid analysis and a lower cost of consumables.

Our results demonstrate that legs are sufficient to distinguish mosquito species using the MALDI-TOF-MS method. The results are available quickly and do not require entomological expertise, for once the spectral reference database is created, it can be transferred and directly used by any laboratory equipped with a MALDI-Biotyper system. The main obstacle for the use of MALDI-TOF-MS is the cost of machine acquisition, but its use thereafter is cost effective [32]. Consequently, we will continue to update our database, including data from our tropical research settings. A MALDI-TOF-MS facility has recently been installed in Dakar, Senegal, through a collaboration between our research team and the microbiology laboratory at the Hospital Principal in Dakar. This facility will be used not only for microbiological purposes but also for ongoing and future entomological surveys to distinguish and identify mosquito vectors in Senegal. Finally, it will be informative to evaluate whether MALDI-TOF-MS can be used to 1) distinguish between uninfected and infected mosquitoes by sporozoites of *Plasmodium* spp. and 2) determine vector resistance to insecticides using protein extracts as samples.

Further research is necessary to establish how useful MALDI-TOF-MS can be in both medical and veterinary entomology, and this technology is highly promising for the fight against mosquito-borne infectious diseases.
